# Neurological complications due to copper deficiency in the context of Wilson disease treatment: a case report with long-term follow-up and review of the literature

**DOI:** 10.1007/s10072-023-07126-8

**Published:** 2023-10-18

**Authors:** Danilo Tornabene, Paola Bini, Matteo Gastaldi, Elisa Vegezzi, Carlo Asteggiano, Enrico Marchioni, Luca Diamanti

**Affiliations:** 1 Department of Brain and Behavioral Sciences, University of Pavia, Pavia, Italy; 2 IRCCS Mondino Foundation, Via Mondino 2, 27100 Pavia, Italy

**Keywords:** Wilson disease, Copper deficiency, Myelopathy, Neuropathy, Copper supplementation, Zinc

## Abstract

The objective is to investigate the presentation, complications, management, and outcomes of copper deficiency-induced neurological pathologies due to Wilson disease (WD) overtreatment. We examined the case of a WD patient who developed a low thoracic dorsal myelopathy due to chronic hypocupremia from excessive zinc therapy. A comprehensive literature review was conducted to identify similar cases. Ten additional cases of neurological pathology resulting from copper deficiency in the context of WD over-treatment were identified, all occurring during therapy with zinc salts. Myelopathy and peripheral neuropathy were the most common complications, while two additional groups reported leukoencephalopathy. Early cytopenia was often associated with copper deficiency-related neurological pathology appearing early in the context of copper deficiency. WD patients undergoing treatment, especially with zinc salts, should be closely monitored to prevent over-treatment and the consequent copper deficiency. Regular complete blood counts could provide early detection of copper deficiency, avoiding irreversible neurological damage. Swift recognition of new neurological signs not consistent with WD and timely discontinuation of the decoppering therapy are critical for improving outcomes. The optimal management, including the potential benefit of copper supplementation in patients with WD and subsequent therapy adjustments, remains unclear and necessitates further investigation. Despite the general poor functional neurological outcomes, there were some exceptions that warrant further exploration.

## Introduction

Copper deficiency (CD) is a rare cause of cytopenia and a form of myeloneuropathy similar to subacute combined degeneration due to vitamin B12 deficiency. The first case of CD-related myelopathy was reported in 2001 [1]. In 2010, a review of 55 published cases of myelopathy due to CD found that the deficiency was most frequently attributed to malabsorption due to gastrointestinal surgery (either bariatric or non-bariatric) or gastrointestinal primary pathology (e.g. celiac disease). Overload of zinc, causing selective copper malabsorption, accounted for nine cases (16%), in turn caused by the use of denture adhesive cream (4 cases), zinc supplements (2 cases), haemodialysis (1 case) or cryptogenic (2 cases) [2]. Myelopathy often appears together with peripheral neuropathy ([3]) and cytopenia (mostly anaemia and/or neutropenia) due to a reversible myelodysplastic syndrome, with haematologic changes usually being the firsts to appear ([4]) and the sole to promptly and completely resolve after CD correction. Neurological deficits, in contrast, tend to show only limited improvement or mere stabilisation with copper supplementation: partial recovery of neurological function was reported in 25% [4] to 49% [2] of cases.

While cases of neurological involvement associated with primary CD have been repeatedly reviewed over the past 20 years, only a few reports are available of iatrogenic cases related to Wilson’s disease (WD) treatment. WD is a genetically determined condition due to ATPB7 biallelic mutations in which impaired biliary excretion of copper leads to its accumulation in the organism and its deposition in several tissues, particularly in the liver (leading to cirrhosis and chronic liver failure) and in the mesencephalon and basal ganglia (producing extrapyramidal motor syndromes with akinesia, rigidity, tremor, ataxia and/or dystonia) [5]. Different patients can develop a predominant involvement of the liver or the brain, especially in the early phases of the disease. The treatment aims to avoid copper accumulation by promoting urinary excretion with copper-chelators (D-penicillamine, trientine) or by inhibiting intestinal absorption of copper with oral zinc salts (zinc induces endogenous chelators in enterocytes, trapping copper in the epithelial cells until desquamation). People with WD are also often advised to follow a low-zinc diet. WD over-treatment can induce CD and thus neurological impairment with a distinct underlying mechanism than primary malabsorption, with unclear pathological implications. Absent specific guidelines and sufficient data from the medical literature, such cases pose significant management issues as copper supplementation can potentially worsen the hepatic and encephalic course of WD without a clear understanding of the potential benefit on the recovery. Long-term follow-up data are also very scarce.

Here we present a case of myelopathy and cytopenia caused by zinc-induced CD in a subject with WD who we have been following for 3 years since the event, and a review of the literature including ten cases of neurological iatrogenic complications due to WD overtreatment. Prevention and management of these complications are discussed.

## Case presentation

We report the case of a 57-year-old Italian woman followed at our centre since the age of 43, when she was diagnosed with WD primarily presenting with neurological signs and symptoms (limb, head and laryngeal dystonic tremor). Ultrasound investigations found signs of subclinical chronic hepatopathy and mild splenomegaly, and no liver failure nor neoplastic lesions were found thereafter. The subject had a familial history of Wilson disease, and her own past medical history included a non-malignant IgG monoclonal gammopathy and stage II chronic kidney disease due to renal angiomyolipomas, treated with ramipril.

After WD diagnosis, a therapy with D-penicillamine (300 mg three times per day) was started, achieving clinical stability, and her tremor was managed for some years with propranolol and botulinum toxin injections into the laryngeal muscles. Due to gastric intolerance to D-penicillamine, after 3 years, the therapy was switched to zinc acetate dihydrate. An initial dose of 150 mg/die of zinc produced an excessive decrease in plasma copper level, so the therapy was continued at a reduced dose of 100 mg/die with satisfactory efficacy.

**Fig. 1:**
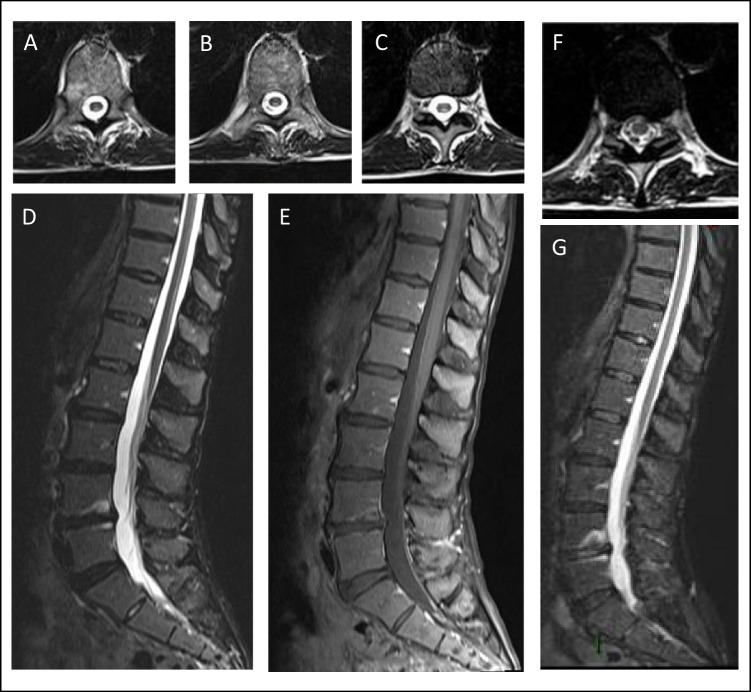
Magnetic resonance imaging: extensive hyperintensity on T2-weighted (**A**, **B**, **C**) and T2-TIRM (**D**) sequences in the dorsal columns of the lower thoracic spinal cord, 10 months after symptoms onset. After gadolinium injection, no contrast enhancement was detected (**E**). One year later, signal abnormalities were no longer visible despite the persistence of clinically significant sensory deficits (**F**, **G**)

At the age of 57, 15 years after receiving the diagnosis of Wilson disease and 12 years after starting zinc therapy, the patient began experiencing paresthesia and dysesthesia in a symmetric sock-like distribution. A comprehensive neurological examination showed lower-limbs hypoesthesia with a distal-to-proximal gradient (most evident for vibration sense), weakness in the dorsal extension of the feet and toes, a positive Romberg sign and gait ataxia. Deep tendon reflexes were normal and the plantar response was downgoing, but some other pyramidal signs (i.e. Oppenheim and Chaddok signs) were observed. Nerve conduction studies proved normal, and the patient was admitted to our Neurology department for further investigations. Upon admission, routine blood exams showed pancytopenia with a mild thrombocytopenia (104.000/mcl), a moderate macrocytic anaemia (Hb 9.4 g/dl, MCV 103.7 fl) and a severe neutropenia (119/mcl). Copper metabolism was evaluated, finding low plasmatic ceruloplasmin levels (2 mg/dl, n.v. 20–60) and low plasmatic copper concentration (21 mcg/dl, n.v. 80–155) but a normal urinary excretion of copper over 24 h (41.8 mcg, n.v. 15–70). Non-ceruloplasmin-bound copper (NCBC, calculated by subtracting from the total copper level 3.15 mcg/dl per 1 mg/dl of ceruloplasmin l [5]) was 14.7 mcg/dl. Blood levels of vitamin B12 and folates were normal at 362 pg/ml and 10.1 ng/ml respectively. Contrast-enhanced magnetic resonance imaging (MRI) only showed previously known brain findings consistent with Wilson disease and a normal spinal cord signal. Cerebrospinal fluid findings were unremarkable. Relevant infectious and autoimmune conditions were ruled out, and bone marrow biopsy was performed before discharge, with normal findings. The cytopenia was therefore attributed to copper deficiency, zinc therapy was discontinued, and the patient was instructed to follow a normal diet, without copper restriction nor supplementation. Neurological findings remained unexplained at discharge, and symptomatic treatment with pregabalin 75 mg b.i.d. was started. Restless leg syndrome was also observed and treated with low-dose pramipexole. After 2 months, MRI follow-up showed dorsal columns hyperintensity in low thoracic spinal cord (T10–T12 level). Neurological examination found a slight worsening of sensory deficits pointing to a spinal cord syndrome (abolished vibration sense at ankles and knees, reduced vibration sense at the superior-anterior iliac spine and a caudal pain hypoestesia with a low dorsal level). Upon review of the relevant literature, the patient was diagnosed with low thoracic myelopathy due to chronic hypocupremia from excessive zinc therapy. After discharge, blood copper levels were checked monthly, and chelating therapy with trientine was introduced after 5 months. Radiologic follow-up found the spinal signal abnormalities to be stable at 6 months from discharge, while the dorsal columns T2 hyperintensities were no longer visible after 16 months (Fig. 1). After 3 years from the onset, only minimal clinical recovery was observed, with persisting gait disability due to sensory ataxia.

## Discussion and review of similar reported cases

A literature search found ten cases of neurological involvement attributed to copper deficiency in the context of WD (over-)treatment, summarised in Table 1. Five cases reported a dorsal columns myelopathy: one cervical isolated myelopathy [6], one cervical myelopathy associated with weakness and myopathic EMG changes [7] and three additional cases of myelopathy (one cervical, two thoracic) associated with sensory (two) [8, 9] or axonal sensory-motor (one) [10] peripheral neuropathy. Three other cases consisted of sensory-motor neuropathy with an especially severe motor involvement, presenting with normal spinal MRI signal but mild somatosensory evoked potentials (SEPs) changes suggesting an associated subclinical spinal involvement [11]–[13]. Lastly, two groups reported CD-induced diffuse brain leukoencephalopathy without mention of spinal or peripheral involvement [14, 15], one of which culminated in a partial status epilepticus refractory to most antiseizure drugs and remitting with CD resolution. Eight out of ten cases presented with mild to severe haematologic anomalies, namely anaemia (macrocytic or normocytic with anisopoikilocytosis) and/or leukopenia (mostly neutropenia), consistent with CD and generally improving rapidly with serum copper level correction. In only one case cytopenia did not improve, and it was ascribed to portal hypertension rather than CD. In addition, one case presented with an associated glomerulopathy and severe albuminuria, with complete resolution after zinc discontinuation [13].

**Table 1:** Ten reported cases of neurological involvement due to Wilson disease overtreatment with available information about management and outcome

Case and authors	Age and sex	Neurological involvement	Haematological findings	Treatment and duration	Copper metabolism	Intervention	Outcome
Herrero et al. 2012 [6]	58 F	Dorsal columns cervical myelopathy (MRI + SEPs +)	Mild macrocytic anaemia (Hb 11.3 mg/dl, MCV 109 fl) and moderate neutropenia (700/mcl with total WBC 3.100/mcl)	Zinc 150 mg/die for 3 years Previously, D-penicillamine for 35 years	Ceruloplasmin: undedectable Serum copper: 3 mcg/dl 24-h urinary copper: undetectable	Zinc discontinuation (later restarted at a lower dose) and oral copper supplementation (dose and duration unspecificed)	Minimal deficit improvement over few months Radiological outcomes not reported Haematological findings remained stable and were attributed to portal hypertension
Wu et al. 2020 [8]	17 F	Dorsal columns upper thoracic myelopathy (MRI +) Sensory axonal neuropathy (EMG/ENG +)	Not reported	Zinc up to 225 mg/die for less than 6 months (did not tolerate penicillamine and trientine, and zinc 150 mg/die was judged not effective enough)	Ceruloplasmin: not reported Serum copper: not reported 24-h urinary copper: significantly diminished (value not reported)	Zinc discontinuation and oral copper supplementation (10 mg/die for 6 months)	Complete clinical recovery Radiological, haematological and EMG/ENG outcomes not reported
Silva-Jùnior et al. 2011 [9]	44 F	Dorsal columns cervical myelopathy (MRI + without gadolinium enhancement) Sensory axonal polyneuropathy (EMG/ENG +)	Macrocytosis (MCV 103 fl) without anaemia, leukopenia (WBC 2.700/mcl, differential count not reported) and mild thrombocytopenia (134.000/mcl)	Zinc 135 mg/die for 14 years (changed after 1 year of penicillamine treatment suspended for tolerance issues)	Ceruloplasmin: 8 mg/dl Serum copper: 3 mcg/dl Calculated NCBC: 0 24-h urinary copper: 7.4 mcg (170 mcg after penicillamine 1 g)	Zinc discontinuation	Neurological symptoms stabilisation (long-term follow-up not reported) Radiological, haematological and EMG/ENG outcomes not reported
Teodoro et al. 2013 [10]	36 M	Dorsal columns thoracic myelopathy (MRI +) Sensory-motor neuropathy (EMG/ENG +)	Moderate anaemia (Hb 8.2 g/dl, MCV not reported)	Zinc 76 mg and trientine 500 mg/die for 16 years	Ceruloplasmin: 3 mg/dl Serum copper: 13.3 mcg/dl Calculated NCBC: 3.85 mcg/dl 24-h urinary cooper: 40.5 mcg Low copper levels in serum (6.35 mcg/dl) and urine (128 mcg/24 h) was observed as early as 5 years before neurological symptoms, with an unspecified worsening in the following years	Trientine discontinuation and zinc reduction to 30 mg/die, later progressively discontinued as well because of persistent copper deficiency	Improvement of ataxia with recovery of ambulatory capability without assistance after 1 year; stability after 1 year from reintroduction of zinc 30 mg/die Mild regression of MRI myelopathy signs after 1 year Slight improvement of sensory action potentials amplitude and conduction velocities after 1 year
Dzieżyc et al. 2014 [7]	37 F	Dorsal columns cervical myelopathy (MRI + SEPs +) Myopathy (EMG +)	Mild leukopenia (2.900/mcl) with normal neutrophils (2.100/mcl)	Zinc 180 mg/die for 16 years	Cerulopasmin: 0.92 mg/dl Serum copper: < 5 mcg/dl Calculated NCBC: < 2.1 mcg/dl 24-h urinary copper: 11 mcg	Zinc discontinuation D-penicillamine introduction after 9 months	Weakness resolution and distal paresthesia improvement Marked improvement of MRI myelopathy signs WBC normalisation after 1 month EMG normalisation SEPs still indicated abnormalities in the dorsal column
Foubert-Samier et al. 2009 [11]	43 M	Sensory-motor neuropathy with severe flaccid distal tetraparesis Impaired tibial SEPs without MRI signs of central nervous system involvement	Mild anaemia (10.6 g/dl, MCV not reported), moderate neutropenia (950/mcl) and moderate lymphopenia (970/mcl), known for 2 years before neurological onset and diagnosed ad myelodysplasia	Zinc 120 mg and trientine 900 mg/die for 28 years	Ceruloplasmin: < 10 mg/dl Serum copper: 3.17 mcg/dl 24-h urinary copper: 108 mcg	Zinc discontinuation and trientine reduction to 300 mg/die (later increased back to 900 mg/die without zinc, after 9 months)	Mild strength improvement after 2 years Rapid haematological findings normalisation Progressive compound action potentials amplitude increment over 2 years, with signs of some motor reinnervation
Cortese et al. 2011 [18]	51 F	Severe length dependent sensory-motor neuropathy (EMG/ENG + with denervation activity) with tetraparesis and severe muscle atrophy Normal MRI of the spine, with minimal SEPs anomalies suggestive of subclinical dorsal column involvement of the spinal cord	Severe neutropenia (250/mcl) and macrocytic anaemia (Hb 6.5 mg/dl, MCV 104.7 fl), slowly developing over 3 years paralleling copper serum level reduction Bone marrow biopsy was suggestive of myelodysplastic syndrome (hypercellularity with trilinear dysplastic changes)	Zinc 276 mg/die for 11 years (previously 138 mg for 12 years) Penicillamine started ad disease onset and discontinued due to hypersensitivity Prior to presentation, the patient had a period of psychogenic anorexia, likely worsening copper deficiency	Ceruloplasmin: not reported Serum copper: 5 mcg/dl 24-h urinary copper: 20 mcg	Zinc reduction to 138 mg/die, then to 50 mg/die Blood transfusions for anaemia	No neurological or EMG/ENG improvement Haematological findings normalisation
Horvath et al. 2010 [13]	40 M	Severe axonal predominantly motor length-dependent neuropathy with tetraparesis and distal muscle wasting (EMG/ENG + with abundant signs of active denervation in distal muscles of four extremities) Normal MRI of the spine, with SEPs anomalies suggestive of dorsal column involvement of the spinal cord	Severe anaemia (7.8 mg/dl, MCV not reported) and neutropenia (260 mg/dl) *This patient also showed glomerulopathy with massive albuminuria, resolving with zinc discontinuation*	Zinc 202 mg/die for 14 years, increased to 253 mg after neuropathy onset (neurological worsening was attributed to Wilson disease progression)	Ceruloplasmin: < 2 mg/dl Serum copper: 3 mcg/dl 24-h urinary copper: 34 mcg	Zinc discontinuation The patient was later kept without decoppering therapy (“until evidence of copper reaccumulation appears”)	Neurological deficits unchanged at 12-month follow-up EMG/ENG outcomes not reported Rapid haematological findings normalisation Albuminuria decrement by 10 times within 3 months and normalisation after 10 months
Benbir et al. 2010 [15]	21 M	Dysarthria and dysphagia for 7 months Partial status epilepticus, refractory to levetiracetam and diphenylhydantoin (responsive to e.v. diazepam) Brain MRI findings: bilateral parieto-occipital subcortical white matter T2-hyperintensity and supratentorial deep white matter atrophy with ventricular dilation. Later, involvement of bilateral putamen, mesencephalon and bilateral middle and superior cerebellar peduncles Spinal of peripheral involvement not reported (clinical, radiological nor neurophysiological)	Not reported	Zinc 100 mg/die, penicillamine 1200 mg/die and strict low-copper diet for 5 years The therapy was increased (dosages not specified) in the 7 months prior to status epilepticus due to neurological disturbances attributed to Wilson disease progression	Ceruloplasmin: not reported Serum copper: 2.2 mcg/dl 24-h urinary copper: 165.3 mcg	Zinc and penicillamine discontinuation and high-copper diet institution Discharged with zinc therapy only (150 mg/die) and a free diet	Gradual seizure cessation within few days Speech and swallowing partial improvement at discharge MRI outcome not reported
Narayan et al. 2006 [14]	17 M	Spastic dysartria, echolalia, occasional outburst of panic, saccadic dysmetria, decreased vibratory and joint position sense in the feet, brisk tendon reflexes, extensor plantar responses, marked truncal ataxia, dystonic posturing of both hands and feet, cogwheel rigidity at the wrists Diffuse deep hemispheric CT hypodensity sparing the posterior lobes, interpreted as demyeliniation, with ventricular dilation and mild cerebral and basal ganglia atrophy Brain MR and spinal imaging were not performed	Normocytic anaemia with anisopoikilocytosis (CBC values not reported)	Zinc 64 mg and penicillamine 750 mg/die for 4 years	Ceruloplasmin: 2.34 mg/dl Serum copper: 16 mcg/dl Calculated NCBC: 8.6 mcg/dl 24-h urinary copper: not reported	Not reported	Not reported

• Cerebrospinal fluid tests, when reported, were always unremarkable.

• Zinc therapy is reported in elemental zinc equivalent dose.

• Normal results for copper metabolism tests are as following: ceruloplasmin 20–35 mg/dl (reduced in Wilson disease, often < 5 mg/dl); total serum copper 60–140 mcg/dl; 24-h urinary copper 20–50 mcg (increased in Wilson disease, often > 100 mcg/dl in subjects not on zinc therapy). Urinary copper is higher on chelating therapy (200–500 mcg/dl) but it should reduce to < 100 mcg after 48 h of therapy cessation (done to assess therapy efficacy and compliance) [5].

• NCPB: Non ceruloplasmine-bound copper, calculated when possible subtracting 3,15 mcg per mg of ceruloplasmin [5].

• MRI + : T2 spinal cord signal hyperintensity in magnetic resonance imaging studies, suggestive of myelopathy. SEPs + : increased conduction latencies in somatosensory evoked potentials study suggestive of slowed central conduction of the nervous signal at spinal cord level. EMG/ENG + : electromyographic and electroneurographic changes suggestive of peripheral neuropathy as described in the “neurological involvement” section (or of myopathy in the case reported by Dzieżyc et al.).

Guidelines on WD advise monitoring of serum and urinary copper as a mean to assess treatment compliance and efficacy, not overtreatment [5, 16, 17]. The European Association for the Study of Liver suggests aiming at a < 100 mcg total copper urinary excretion during zinc therapy to prove efficacy without specifying a lower reference limit, while guidelines from the American Association for the Study of Liver Disease and from the European Society for Paediatric Gastroenterology, Hepatology and Nutrition give a desirable range of 30 to 75 mcg/24 h, thus advising a stricter level of decoppering but providing a lower reference. For patients on chelating therapy reference values for urinary copper are 200–500 mcg/24 h, with a target of < 100 mcg 48 h after cessation of chelating treatment for scheduled testing. Normal values for total serum copper are 60 to 140 mcg/dl. However, NCBC is sometimes suggested as a better index for treatment monitoring in Wilson disease when ceruloplasmin values are reduced and NCBC < 15 mcg/dl is considered as appropriate in treated subjects by all guidelines, but no lower end reference is provided to detect overtreatment.

In the cases we reviewed, the total copper level, when reported, was always low (16 mcg/dl and 13.3 mcg/dl in two cases, ≤ 5 mcg/dl in seven others). NCBC was inferable in four cases, one at 8.6 mcg/dl and three ≤ 3.85 mcg/dl. When a 24-h urinary copper was reported, it was generally low as well in patient treated with zinc monotherapy, with two exceptions (34 mcg, 20 mcg, 11 mcg and 7,4 mcg in four cases, “undetectable” in a fifth and unspecified but “significantly reduced” in a sixth). Three subjects taking a combination of zinc and a chelating agent (increasing urinary over biliary excretion of copper) had higher excretion levels, but still lower than the recommended 200 mcg/24 h (165 mcg, 108 mcg and 40 mcg).

In the case we report, total serum copper was indeed low (21 mcg/dl) but this finding seemed to be related to the very low ceruloplasmin concentration in blood, as 24-h urinary copper and calculated NCBC were both normal at 41.5 mcg and 14.7 mcg/dl respectively. However, the coexistence of a monophasic dorsal column myelopathy, macrocytic anaemia and severe neutropenia in a patient chronically treated with zinc with normal vitamin B12, vitamin E and folates level and unremarkable bone marrow findings is suggestive of CD, especially when considering the rapid resolution of cytopenia we observed after zinc discontinuation. Measured copper values could theoretically be wrong due to analytic errors, or they could be not sensitive enough as markers of copper deficiency. This case, however, demonstrates the importance of haematologic findings when evaluating a subject with Wilson disease complaining of new neurological disturbances, and in general in monitoring of patients on zinc therapy.

In at least two reported cases [11, 18], cytopenia was known years before neurological symptoms onset, and a diagnosis of myelodysplasia was made without changes in the decoppering therapy; intervention at that point would presumably have avoided irreversible neurological deficit. In two additional cases [13, 15], new neurological signs and symptoms were interpreted as a worsening of WD itself, and decoppering therapy was increased for several months before recognising CD as the cause and discontinuing the culprit drug. Presumably, in both cases, a simple complete blood count (CBC) could have shown cytopenia prompting zinc dose adjustment before irreversible neurological damage.

Distinguishing neurological worsening due to copper deficiency (i.e. overtreatment) rather than to copper accumulation (i.e. undertreatment) can prove difficult, especially when the main physician managing the case is not a neurologist. Myelopathy and peripheral neuropathy could be suspected based on a thorough neurological examination as sensory disturbances, deep tendon reflexes changes and amyotrophy would not be expected in a classical extrapyramidal WD pathology. Disturbances as in *Benbir *et al*.* (dysarthria and dysphagia) [15] could, however, theoretically be caused by WD, and the haematologic findings from a CBC could represent an easily available discriminative factor (although the authors do not report any).

In addition to routine serum and urinary copper measurement advised for efficacy and compliance monitoring, thus, WD patients could potentially benefit from regular CBC for early detection of possible iatrogenic effects. This appears to be especially important in subjects treated with zinc, as all of the reported CD complication happened during zinc therapy (either as monotherapy or in combination with chelating therapy). Due to a better perceived tolerability compared with chelating agents, zinc is often used in the chronic phase of the disease as a life-long treatment, when the patients are more likely not to be followed as strictly as in the first years after the diagnosis [5]. It was even proposed zinc itself could be neurotoxic [19]; this suggestion, however, does not appear convincing to us, considering the similarities between myeloneuropathy in zinc-treated WD patients and the ones occurring in other zinc-deficient subjects due to other malnutrition conditions (e.g. bariatric surgery or celiac disease), and the reversible haematological changes similar to the ones known to be caused by CD.

### Management and outcome

Another concern is the best management strategy of such cases of neurological iatrogenic consequences. Normally, selective malnutrition would be treated with supplementation of the specific micronutrient involved, but many clinicians faced with this situation felt that administering copper to a person with WD was not advisable and only decided to discontinue the ongoing decoppering therapy (either gradually or abruptly) and to encourage a free diet (i.e. not copper-free). In one case in which copper supplementation was given (10 mg/die p.o. for 6 months), the patient, a 17-year old woman with a myelopathy, achieved a good clinical recovery [8]. This patient, however, had been on decoppering therapy for 6 months only, as opposed to the many years of treatment reported in all of the other cases, and could then have theoretically been in a milder or shorter copper depletion state. The report of another subject, who developed both a myelopathy and a sensory neuropathy and received oral copper supplementation after 3 years of zinc therapy and a total of 38 years of decoppering therapy, describes only minimal improvements of the symptoms over the first few months, with a stabilisation later [6]. Other cases were managed without copper supplementation. Amongst the other three reported cases with a prominent myelopathy are two reported partial improvement [7, 10] and one a mere stabilisation [9]. Of the three subjects with severe motor neuropathy, only one showed a mild clinical improvement after 2 years of follow-up [11]. The two reports of subjects showing leukoencephalopathy did not include any follow-up, but in one case, therapy discontinuation achieved seizure cessation and improvement of dysarthria and disfagia to the point where the patient was discharged with zinc therapy (at a lower dose than the one which produced the symptoms) [15]. In our case, where myelopathy was the only observed neurological involvement and management included zinc discontinuation without copper supplementing, with subsequent trientine introduction after 5 months, only stabilisation of the deficits was achieved without significant clinical improvement after 3 years (which is the longest follow-up available in literature), despite resolution of MRI spinal changes after 16 months. Overall, satisfactory clinical improvement of the neurological disturbances seems rare in these cases, prompt recognition of CD and decoppering therapy discontinuation appear important to stop the neurological degeneration and favour recovery, and zinc supplementation might have a role in improving the chance of a good functional outcome of the CD-induced myeloneuropathy (although it can potentially worsen the course of the underlying WD in patients not on therapy).

In any case, WD patients developing CD-related complication and discontinuing decoppering treatment will need to restart a therapy at some point, and how exactly this is done can be a challenging decision. In our case, the patient was kept without therapy and on a free diet for 1 year with a close neurological, hepatological, haematological and metabolic follow-up, and then it was decided by the patient’s hepatologist to restart decoppering with a different drug (trientine). Other patients restarted zinc at a lower dose than the one which induced CD [6, 15, 18]. Available data are not enough to inform on the best course of action, but frequent monitoring of CBC, serum and urinary cooper, hepatic function and imaging and neurological function (both to capture any improvement and to detect any worsening due brain copper deposition) appear essential to establish the timing for decoppering therapy reintroduction. No recurrence of any CD-related neurological damage has been reported so far after therapy reinstitution.

## Conclusions

Patients treated for WD can develop a range of neurological complications from iatrogenic CD, including dorsal column myelopathy, peripheral neuropathy and leukoencephalopathy. Treated patients should therefore be monitored to detect over-treatment, especially when on zinc and in the chronic phase of WD stability, but this need might be underestimated. Early cytopenia of variable severity is normally associated with CD-related neurological involvement. Regular CBCs, in addition to direct copper measurement in urine and blood, are a simple tool which appears useful in detecting significant CD before irreversible neurological damage, and the appearance of any cytopenia should prompt a revaluation of the therapy.

What the optimal management of these cases should be is unclear, and copper supplementation might have a role. Functional neurological outcome seems generally poor, with some significant exception. Prompt recognition of new neurological signs and symptoms not consistent with WD is crucial to improve the outcome by discontinuing the decoppering therapy as soon as possible. After CD resolution, CBC normalisation and neurological stabilisation, switching to chelating therapy (as opposed as continuing with zinc therapy) could be safer, if tolerated, although no recurrence of CD-related iatrogenic complications has been reported yet. It is unclear how long the patient should be kept off therapy to allow neurological recovery, as the possibility of a recovery itself is uncertain and probably unlikely in most cases.
